# Surgical Treatment Options for Geriatric Patients With Benign Prostatic Hyperplasia: Advocating for Early Transdisciplinary Geriatric Involvement and Minimally Invasive Surgical Options

**DOI:** 10.7759/cureus.111290

**Published:** 2026-06-22

**Authors:** Yousef E Abu Osba, Sara B Alrafayia

**Affiliations:** 1 Urology, University Hospitals of Leicester NHS Trust, Nottingham, GBR; 2 Obstetrics and Gynecology, Nottingham University Hospitals NHS Trust, Nottingham, GBR

**Keywords:** benign prostatic hyperplasia (bph), comprehensive geriatric assessment (cga), elderly patient care, lower urinary tract symptoms (luts), minimally invasive surgical procedures

## Abstract

Benign prostatic hyperplasia (BPH) is highly prevalent among ageing men and frequently results in lower urinary tract symptoms (LUTS) that significantly impair quality of life. With increasing life expectancy, clinicians are increasingly required to manage elderly patients with complex comorbidities, frailty, and reduced physiological reserve. While pharmacological therapy remains the first-line treatment, surgical intervention is often required for refractory or complicated disease.

Decision-making in this population extends beyond traditional surgical considerations and requires a holistic, patient-centered approach. This narrative review has two primary aims. First, it examines contemporary surgical treatment options for BPH in elderly patients, integrating current evidence on efficacy and safety. Second, and more critically, it advocates minimally invasive surgery as a viable and underutilized alternative to long-term urinary catheterization in patients with multiple comorbidities who may not be suitable candidates for conventional surgery. In addition, it supports the early involvement of transdisciplinary geriatric medicine teams in the development of management plans and the selection of surgical strategies. Incorporating comprehensive geriatric assessment (CGA) into routine practice may improve risk stratification and better align treatment with patient-centered outcomes, such as functional independence and quality of life.

## Introduction and background

Epidemiology and clinical impact

Benign prostatic hyperplasia (BPH) is a common urological condition that becomes increasingly prevalent with age. Studies have shown that nearly 50% of men over the age of 60 years, and up to 90% of men over the age of 80 years, have evidence of prostatic hyperplasia [1,2]. This may manifest as lower urinary tract symptoms (LUTS), urinary retention, recurrent urinary tract infections (UTIs), or renal impairment.

BPH is a non-malignant enlargement of the prostate gland that may result in compression of the prostatic urethra and subsequent bladder outlet obstruction (BOO). This commonly manifests as lower urinary tract symptoms (LUTS), including urinary frequency, nocturia, urgency, hesitancy, weak urinary stream, and a sensation of incomplete bladder emptying [1,2]. Moreover, LUTS can adversely affect quality of life, sleep, and independence. This is particularly significant in elderly men, the majority of whom experience at least one LUTS. These symptoms may contribute to falls, cognitive decline, and social isolation, highlighting the impact of BPH beyond the urological system alone [3].

The challenge of surgical decision-making in the elderly

Deciding on the most appropriate surgical intervention for elderly patients may present a unique clinical challenge. Elderly patients commonly have multiple comorbidities, polypharmacy, frailty, and reduced physiological reserve [4,5], all of which may influence perioperative risk and postoperative recovery.

However, age alone should not determine treatment eligibility. Modern geriatric medicine emphasizes the need for a comprehensive assessment when considering treatment options, including evaluation of functional status, quality of life, cognition, and life expectancy [5]. This is particularly relevant in the management of BPH, where a wide range of surgical interventions is available.

Aim of the review

This review has two central aims. The first is to provide a comprehensive overview of surgical treatment options for BPH in elderly patients while advocating for the early and routine involvement of transdisciplinary geriatric medicine teams in management planning [6,7]. The second is to advocate for minimally invasive surgical procedures as a meaningful clinical alternative to long-term urinary catheterization in high-risk patients, given the well-documented burden of catheter-related complications. Integrating geriatric assessment principles into surgical decision-making enables clinicians to better individualize treatment selection, optimize perioperative care, and prioritize outcomes that are most relevant to older adults, including independence and quality of life [7].

Methods

A narrative search of PubMed, Google Scholar, and MEDLINE was performed using terms including "benign prostatic hyperplasia", "elderly", "frailty", "TURP", "HoLEP", "Rezūm", "UroLift", "prostatic artery embolization", and "comprehensive geriatric assessment", supplemented by relevant EAU and NICE guidelines. Preference was given to studies published within the last 10 years, with older landmark studies included when they provided foundational evidence. Articles were selected based on their relevance to the geriatric population, methodological quality, and clinical applicability.

Role of geriatric medicine and transdisciplinary care

The geriatric population is expanding worldwide. In Europe, it is estimated that 28% of the population will be aged ≥65 years by 2050, with a significant proportion aged over 85 years [4]. Consequently, clinicians are increasingly encountering older men with symptoms of BPH requiring treatment.

Comprehensive geriatric assessment (CGA) has become the gold standard for evaluating older adults, incorporating a multidimensional assessment of functional capacity, cognition, nutrition, comorbidities, and frailty [5,7]. Although geriatric medicine has become increasingly developed and emphasizes a holistic approach and multidisciplinary team (MDT) involvement in the care of older adults [5], the integration of these principles into the management of geriatric patients with surgical conditions remains less well defined.

The elderly surgical patient presents unique challenges due to the interaction between physiological ageing, comorbidity burden, and psychosocial factors. These complexities necessitate a transdisciplinary approach involving geriatricians, urologists, anesthetists, and allied health professionals [7]. Early geriatric involvement facilitates risk stratification, optimization, and shared decision-making, ultimately improving perioperative outcomes and preserving functional independence [7].

Urinary symptoms in older adults frequently coexist with incontinence, functional impairment, skin complications, falls risk, and caregiver burden, all of which extend beyond the traditional scope of urological assessment. For this reason, involvement of a continence team as part of CGA may further strengthen care planning in elderly patients with BPH. Continence-focused input can help identify reversible contributors, optimize bladder management strategies, support skin care and toileting plans, and align treatment decisions with functional goals and quality-of-life priorities. This is particularly relevant in frail older adults, in whom urinary symptoms often reflect multidimensional geriatric syndromes rather than isolated lower urinary tract pathology [8].

Pathophysiology of BPH

BPH is defined as the benign proliferation of epithelial and stromal cells within the transitional zone of the prostate. This may or may not result in BOO, leading to LUTS. Multiple factors and mechanisms contribute to the development of BPH.

Hormonal Factors

Androgens, particularly dihydrotestosterone (DHT), play a central role in the proliferation of prostatic epithelial cells. DHT is formed within the prostate through the action of the enzyme 5-alpha-reductase, which converts testosterone to DHT. Elevated DHT levels stimulate stromal and epithelial proliferation, resulting in prostatic enlargement [9]. Age-related changes in androgen metabolism, together with a relative increase in estrogen levels, may further promote prostatic hyperplasia [10].

Inflammation and Cellular Senescence

Chronic inflammation within the prostate is also recognized as a contributor to BPH. Histological studies have shown that infiltration by T lymphocytes, macrophages, and cytokine-mediated pathways contributes to tissue remodeling in hyperplastic prostates [11]. In older patients, low-grade chronic inflammation may accelerate stromal proliferation and worsen LUTS, regardless of prostatic size [11].

Bladder and Lower Urinary Tract Changes

With ageing, the structure and function of the bladder may change. Decreased detrusor contractility and compliance, together with altered sensory perception, contribute to incomplete bladder emptying and worsening storage symptoms [3]. Elderly men often present with a mixed pathology comprising BOO secondary to BPH and concomitant detrusor underactivity, which may complicate treatment outcomes.

Comorbidity Interactions

Multiple comorbidities are commonly encountered in older men presenting with LUTS, including diabetes, cardiovascular disease, neurological disorders, and cognitive impairment, all of which may exacerbate urinary symptoms. Moreover, polypharmacy can complicate the management of LUTS. For example, alpha-agonists and anticholinergic medications may increase post-void residual volume and precipitate urinary retention [5].

## Review

Indications for surgical intervention in geriatric patients

In cases where conservative or medical management of BPH fails, surgical intervention may be indicated. Examples of indications for surgery include moderate-to-severe LUTS that do not respond to medical therapy, urinary retention, recurrent UTIs, bladder stones, and patient preference [1,12].

Early involvement of geriatric medicine is particularly valuable at this stage, allowing for comprehensive assessment and alignment of treatment decisions with patient goals and functional priorities [7]. This approach facilitates careful balancing of the anticipated benefits of surgery against perioperative risk, while taking into account the patient's comorbidities, frailty, cognitive status, and life expectancy [5,13].

NICE guidance recommends reserving surgical intervention for men with severe voiding symptoms or for those in whom conservative and pharmacological therapies have failed or are inappropriate. The guidance also emphasizes the importance of shared decision-making and discussion of alternative treatment strategies prior to operative intervention [14].

Complications of long-term urinary catheterization

For elderly men with BPH deemed unsuitable for conventional surgery, long-term indwelling urinary catheterization is frequently employed as a default management strategy. However, this approach carries a substantial and well-documented burden of complications that is often underappreciated in clinical decision-making, and it should not be accepted as a passive alternative when minimally invasive surgical options exist.

Catheter-associated UTI (CAUTI) remains one of the most common complications of long-term catheterization and accounts for approximately 40% of all nosocomial infections [15]. However, the reported incidence varies considerably depending on the patient population, catheter duration, and surveillance definitions [16].

Surveys across 12 European countries indicate that 5.4% of people aged over 65 years live with long-term indwelling urinary catheters, with approximately 90,000 long-term catheter users in the UK alone [17]. Age-related degeneration of the urinary tract mucosa and decline in local antibacterial defenses significantly increase susceptibility to infection in this population [17]. Urosepsis may result from uncontrolled UTI, and mortality has been reported to be more than three times higher in catheterized than in non-catheterized individuals [16].

Beyond infection, long-term catheterization in men is associated with urethral damage, epididymitis, orchitis, scrotal abscess, prostatitis, prostatic abscess, urethral stricture formation, traumatic hypospadias, and periurethral fistula [15]. Catheter blockage due to encrustation and biofilm formation is a recurring problem, leading to bypassing, catheter changes, and unplanned healthcare contact. Bladder stones may develop with prolonged use, and there is a recognized long-term association between permanent catheterization and bladder malignancy, although the absolute risk remains low and is generally observed only after prolonged periods of chronic catheter use [16].

The impact on quality of life is equally significant. Catheter-related complications, including bladder spasms, discomfort, leakage, and recurrent infections, can significantly impair daily activities, sleep, mobility, and social participation [17]. Indwelling catheters are also associated with loss of independence, reduced ability to engage in sexual activity, and psychological distress, all of which are particularly detrimental in a geriatric population in which functional preservation is a primary treatment goal [17]. This body of evidence strongly supports active consideration of minimally invasive surgical alternatives as catheter-sparing options for high-risk elderly patients, rather than defaulting to long-term catheterization.

Surgical treatment options

Transurethral Resection of the Prostate (TURP)

TURP has long been considered the gold standard surgical treatment for BPH [1]. The procedure uses a transurethral resectoscope with electrocautery to remove obstructing prostatic tissue, thereby relieving bladder outlet obstruction. It is supported by decades of long-term data demonstrating significant improvements in IPSS scores, urinary flow rates, and quality of life, with favorable outcomes in patients with moderate-sized prostates. In the geriatric population, TURP carries recognized perioperative risks, including bleeding and TUR syndrome, which results from the absorption of hypotonic irrigating fluid and may lead to hyponatremia and cardiovascular complications. Patients with cardiovascular comorbidities may also have increased anesthetic risk, making careful fluid management and electrolyte monitoring essential throughout the perioperative period [13,18]. The introduction of bipolar diathermy, which allows resection using isotonic saline irrigation, has substantially reduced the risk of TUR syndrome. In addition, shorter operative times achievable with modern equipment may reduce overall anesthetic exposure in frail patients. Studies have confirmed significant improvements in LUTS and urinary flow in elderly patients, although perioperative geriatric assessment and optimization remain essential components of safe management in this population [13,18].

Laser-Based Procedures

Laser-based procedures, principally holmium laser enucleation of the prostate (HoLEP) and photoselective vaporization of the prostate (PVP), have become increasingly prominent, particularly for patients receiving anticoagulation therapy or those with larger prostates [19,20]. These procedures offer a substantially lower risk of bleeding than conventional TURP, making them especially relevant for elderly patients receiving antithrombotic therapy, in whom interruption of anticoagulation may carry significant risks. Hospital stays are generally shorter, catheter removal is earlier, and long-term efficacy outcomes are comparable to those of TURP. For prostates exceeding 80 mL in volume, HoLEP is particularly effective and is considered size-independent in its applicability. The primary limitation is the requirement for experienced surgeons, as HoLEP has a well-recognized learning curve. Despite this, laser-based procedures are increasingly being positioned as the preferred surgical option for geriatric patients with multiple comorbidities, and evidence supporting their use in this population continues to grow [19,20].

Open and Robotic-Assisted Prostatectomy

Open prostatectomy is reserved for patients with very large prostates (typically > 80-100 mL) or concomitant bladder pathology requiring simultaneous surgical management [1]. It allows effective removal of large adenomas and can address concurrent bladder or urethral pathology during the same operative setting. However, open surgery is associated with higher morbidity and a prolonged recovery period, making it less suitable for frail elderly patients. Robotic-assisted simple prostatectomy represents an emerging alternative that reduces intraoperative blood loss and shortens hospital stay compared with open surgery, although it still requires general anesthesia and access to specialized robotic platforms [21]. Suitability in the geriatric population depends critically on physiological age, cardiopulmonary reserve, and frailty status rather than chronological age alone. Evidence suggests that carefully selected, fit elderly patients can achieve excellent symptomatic relief and durable outcomes; however, a thorough risk-benefit assessment is essential before proceeding [6,22].

Prostatic Artery Embolization (PAE)

PAE is a minimally invasive endovascular procedure performed by interventional radiologists that aims to reduce prostatic blood supply, leading to ischemia and sustained reduction in prostate volume [23,24]. It can be performed under local anesthesia on an outpatient basis, thereby avoiding the need for general or spinal anesthesia, which makes it an attractive option for patients who are not suitable surgical candidates because of high anesthetic risk. Perioperative risks and complications are generally low, and evidence supports its use in high-risk patients with significant comorbidities [21,23]. The limitations of PAE must nonetheless be acknowledged. Long-term data remain less robust than those for TURP and HoLEP, repeat procedure rates are higher, and the technique requires specialist interventional radiology expertise that is not universally available. It is therefore best considered as part of a multidisciplinary discussion in carefully selected patients rather than as a routine surgical alternative.

Rezūm Water Vapor Therapy

Rezūm water vapor therapy is a minimally invasive procedure that uses convective thermal energy delivered via sterile water vapor injected trans-urethrally into the prostatic adenoma. The heat disrupts prostatic cell membranes, leading to cell death and progressive tissue resorption over several weeks, resulting in sustained reduction of prostatic obstruction [25]. The procedure can be performed under local anesthesia, with intravenous sedation in an outpatient setting, requiring no general anesthesia, which makes it particularly well suited to elderly patients with high anesthetic risk [25].

Evidence supporting the use of Rezūm in very elderly patients is growing. A multicenter cohort study of patients aged 80 years and older demonstrated that Rezūm was successfully performed under propofol-based intravenous sedation in 72.4% of cases as a day-case procedure, with no major cardiovascular or thrombotic events despite a high comorbidity burden, including hypertension, diabetes, and widespread antithrombotic therapy [26]. Significant improvements in IPSS scores and quality of life were sustained at two-year follow-up. Importantly, 20.7% of patients in that cohort were catheter-dependent prior to the procedure, and Rezūm successfully facilitated catheter independence, directly addressing one of the most debilitating consequences of undertreated BPH in the geriatric population [25]. A systematic review has further supported the use of Rezūm in patients with urinary retention and permanent catheter dependence secondary to BPH, reinforcing its role as a catheter-sparing intervention [25]. Five-year data from randomized controlled trials have demonstrated durable improvements in IPSS, urinary flow rates, and quality of life, with low rates of erectile and ejaculatory dysfunction, a clinically important advantage over TURP in a population in which sexual function and continence remain relevant quality-of-life domains [26]. Rezūm is also suitable for prostates with a median lobe component, extending its applicability beyond that of UroLift. For high-risk elderly patients in whom conventional surgery is inadvisable, Rezūm provides a clinically meaningful pathway to symptom relief and catheter independence [25-27].

UroLift (Prostatic Urethral Lift)

UroLift is a minimally invasive mechanical procedure in which permanent nitinol and stainless-steel implants are deployed endoscopically to retract the lateral lobes of the prostate, thereby widening the urethral lumen without removing, cutting, or ablating tissue [28]. It can be performed under local anesthesia in an outpatient setting, with rapid recovery, minimal blood loss, and preservation of ejaculatory function, characteristics that are highly relevant to elderly patients wishing to avoid general anesthesia and prolonged recovery [28,29]. The L.I.F.T. trial and its three- and five-year follow-up data have demonstrated sustained improvements in IPSS, quality-of-life scores, and peak urinary flow rate [28]. Compared with TURP, UroLift demonstrates comparable quality-of-life outcomes, with superior recovery time and preservation of ejaculatory function [12]. However, UroLift is not appropriate for patients with a significant median lobe, and retreatment rates are higher than those associated with more definitive procedures such as TURP or HoLEP [28,29]. It is best positioned as a catheter-sparing option in selected patients with moderate symptoms and no significant median lobe, particularly those who prioritize preservation of sexual function and rapid return to normal activity.

Summary of surgical options

The diversity of available surgical options for BPH necessitates a structured approach to selecting the most appropriate procedure for each patient. Table 1 summarizes the six main surgical modalities discussed in this review, comparing them in terms of anesthetic requirements, advantages, limitations, and suitability for different patient groups.

**Table 1:** Summary of surgical options HoLEP: holmium laser enucleation of the prostate, PVP: photoselective vaporization of the prostate, TURP: transurethral resection of the prostate. PAE: prostatic artery embolization, GA: general anesthesia.

Procedure	Anesthesia	Key advantages	Key limitations	Best suited for
TURP	General/spinal	Gold standard; long-term data; effective for moderate prostates [1]	Bleeding risk; TUR syndrome; higher anesthetic burden [13,18]	Fit elderly; moderate prostate size [13,18]
HoLEP/PVP	General/spinal	Lower bleeding; size-independent (HoLEP); durable outcomes [19,20]	Learning curve; requires an experienced surgeon [19,20]	Patients on anticoagulation; large prostates [19,20]
Open/robotic prostatectomy	General	Effective for very large prostates; addresses concurrent pathology [1,21]	Highest morbidity; prolonged recovery [21]	Selected fit patients with very large prostates (>100 mL) [21]
PAE	Local	No GA; outpatient; suitable for high anesthetic risk [23]	Limited long-term data; higher retreatment; needs IR expertise [23,24]	Very high-risk patients; not surgical candidates for conventional surgery [23,24]
Rezūm	Local/IV sedation	No GA; outpatient; catheter-sparing; suitable for median lobe [25,26]	Short-term catheter post-procedure [27]	High-risk elderly; catheter-dependent patients; high comorbidity burden [25]
UroLift	Local	No GA; rapid recovery; preserves ejaculatory function [28,29]	Not suitable for median lobe; higher retreatment rate [28,29]	Moderate symptoms; sexually active; no median lobe [28,29]

Anesthesia considerations in geriatric BPH surgery

Part of the decision-making process for selecting the most appropriate surgical option for geriatric patients relates to the type of anesthesia required and the associated physiological strain. Age-related physiological changes, comorbidities, and frailty increase susceptibility to anesthetic complications. Frailty scores and organ reserve should be taken into account to guide the choice of anesthesia [5]. Regional anesthesia (spinal or epidural) reduces systemic stress, cardiovascular complications, and postoperative delirium, and should be preferred where feasible [22]. General anesthesia may be necessary for longer or more complex procedures; however, a thorough cardiovascular and pulmonary assessment is required beforehand [22]. Perioperative optimization includes fluid management, review of regular medications, including blood thinners and antihypertensives, and delirium prevention strategies [22]. Minimally invasive procedures performed under local anesthesia or minimal sedation, such as Rezūm, UroLift, and PAE, are generally preferred in high-risk geriatric patients, and their availability should be actively considered before proceeding with conventional surgery under general anesthesia.

Postoperative outcomes and complications

Despite these risks, well-selected elderly patients experience substantial improvements in LUTS, urinary flow, and quality of life across all major surgical modalities [13,21,23]. Elderly patients undergoing BPH surgery are at higher risk of complications, including intra- and postoperative bleeding (higher with TURP and lower with laser-based procedures), infection and sepsis, delirium and cognitive dysfunction (more common in frail and cognitively impaired patients), and cardiopulmonary complications, including arrhythmias, hypotension, and electrolyte imbalance. Urinary incontinence and erectile dysfunction are usually transient but should be discussed with patients during counselling [13,20,24].

Discussion

Current approaches to BPH management remain largely procedure-driven, focusing on symptom severity and anatomical factors such as prostate size, while failing to adequately account for the heterogeneity of ageing, including frailty, cognitive function, functional status, and patient-centered goals, which fundamentally shapes outcomes in the geriatric population [1,5,7,12]. Traditional risk assessment tools often underestimate the impact of geriatric syndromes, which are strong predictors of postoperative morbidity and mortality [7,30]. Early integration of geriatric medicine into the surgical decision-making process enables a meaningful shift from a procedure-centered to a patient-centered model of care, with CGA facilitating identification of modifiable risk factors, optimization of comorbidities, and alignment of treatment with patient goals [7,31]. Evidence from perioperative geriatric models demonstrates improved outcomes, including reduced complications and improved functional recovery, when geriatric teams are involved early [6,7]. A phenotype-driven approach to surgical selection, incorporating frailty and functional status, allows for more appropriate matching of patients to interventions: robust patients may benefit from definitive procedures such as TURP or HoLEP, while frail or high-risk patients may derive greater and safer benefit from minimally invasive approaches such as Rezūm, UroLift, or PAE [7,22,23,30]. Functional outcomes, including mobility, independence, and cognitive trajectory, are frequently overlooked in BPH surgical literature; yet evidence from geriatric surgery suggests that even minor procedures can precipitate functional decline in vulnerable patients, underscoring the importance of incorporating these metrics into decision-making [6,30]. Postoperative delirium remains a common and serious complication in elderly patients, associated with increased morbidity and mortality, and its prevention requires geriatric-informed perioperative strategies [7,22]. At a system level, barriers remain, including limited access to geriatric services and a lack of integrated care pathways, and addressing these requires organizational change and increased cross-specialty collaboration [7,32]. Future models of care should incorporate early CGA, frailty-based stratification, and collaborative decision-making within a transdisciplinary framework, approaches that align with the evolving field of geriatric surgery and have the potential to improve outcomes and optimize resource utilization [6,7,32].

Future directions

Future research should focus on integrating geriatric assessment tools, including validated frailty indices, into prospective BPH surgical trials, developing predictive models that incorporate frailty alongside traditional urological outcome measures, and evaluating functional and patient-reported outcomes following BPH surgery in very elderly patients. Comparative effectiveness data between minimally invasive procedures (Rezūm, UroLift, and PAE) in high-risk geriatric populations are particularly needed, as is cost-effectiveness analysis of transdisciplinary perioperative care models [7,26].

## Conclusions

BPH is highly prevalent in elderly men and frequently requires surgical management; however, optimal care in this population extends well beyond procedural selection. This review has highlighted two interconnected principles that should guide clinical practice. First, minimally invasive surgical procedures, particularly Rezūm water vapor therapy, represent a clinically important and underutilized alternative to long-term urinary catheterization in elderly patients with multiple comorbidities who are not suitable candidates for conventional surgery. The burden of long-term catheterization, including recurrent infection, urosepsis, urethral damage, and profound impairment of quality of life, makes it an outcome that should be actively avoided when surgical alternatives exist. Rezūm has demonstrated safety, efficacy, and the capacity to restore catheter independence, even in patients aged 80 years and older with a high comorbidity burden. Second, the routine and early involvement of transdisciplinary geriatric medicine teams in the management of elderly patients with BPH is essential for achieving outcomes that matter most to this population. Incorporating geriatric principles into surgical pathways, through CGA, frailty-based risk stratification, and shared decision-making, enables personalized care, improved perioperative outcomes, and preservation of independence and quality of life. Age alone should never determine surgical eligibility; rather, every decision should be guided by a comprehensive, patient-centered assessment.

## References

[1] Kaplan SA (2004). AUA guidelines and their impact on the management of BPH: an update. Rev Urol.

[2] Roehrborn CG (2005). Pathology of benign prostatic hyperplasia. Prostate.

[3] Papanikolaou D, Diamantopoulos C, Sokolakis I (2026). The role of the aging bladder in lower urinary tract symptoms: pathophysiology and therapeutic implications in patients with benign prostatic hyperplasia. Medicina (Kaunas).

[4] Grundy EM, Murphy M (2020). Population ageing in Europe. Oxford Textbook of Geriatric Medicine.

[5] Cesari M, Arosio B (2022). The ageing bladder. Pathy's Principles and Practice of Geriatric Medicine.

[6] Ellis G, Gardner M, Tsiachristas A (2017). Comprehensive geriatric assessment for older adults admitted to hospital. Cochrane Database Syst Rev.

[7] Partridge JS, Harari D, Dhesi JK (2012). Frailty in the older surgical patient: a review. Age Ageing.

[8] Davis NJ, Wyman JF, Gubitosa S, Pretty L (2020). Urinary incontinence in older adults. Am J Nurs.

[9] Schröder FH, Blom JH (1989). Natural history of benign prostatic hyperplasia (BPH). Prostate Suppl.

[10] David BG (2025). The role of hormonal changes in the development of benign prostatic hyperplasia: a mini review. NIJPP.

[11] Sciarra A, Mariotti G, Salciccia S, Autran Gomez A, Monti S, Toscano V, Di Silverio F (2008). Prostate growth and inflammation. J Steroid Biochem Mol Biol.

[12] Reich O, Gratzke C, Stief CG (2006). Techniques and long-term results of surgical procedures for BPH. Eur Urol.

[13] Ratanatherawichian R, Preechakoon B, Pungkate P, Intarakaew T, Vichitvejpaisal P (2017). Perioperative nursing considerations for transurethral resection prostatectomy. Curr Opin Urol.

[14] (2019). National Institute for Health and Care Excellence. Lower urinary tract symptoms in men: management. http://www.nice.org.uk/guidance/cg97.

[15] (2023). UroToday. Complications - indwelling urinary catheters. https://www.urotoday.com/urinary-catheters-home/indwelling-catheters/complications/problems.html#references.

[16] Sørbye LW, Finne-Soveri H, Ljunggren G, Topinková E, Bernabei R (2005). Indwelling catheter use in home care: elderly, aged 65+, in 11 different countries in Europe. Age Ageing.

[17] Christiaans CH, van Veen FE, Scheepe JR, Blok BF (2025). Patient satisfaction, quality of life, and catheter-related complications in long-term urinary catheter users: a nationwide survey. World J Urol.

[18] Wang K, Chen M, Liu Y, Xiao W, Qian Y, Liu X (2022). Efficacy and safety of prostatic artery embolization in the treatment of high risk benign prostatic hyperplasia and its influence on postoperative life quality of patients. Front Surg.

[19] de la Rosette J, Collins C, Bachmann A (2008). Historical aspects of laser therapy for benign prostatic hyperplasia. Eur Urol Suppl.

[20] Sun F, Yao H, Bao X (2022). The efficacy and safety of HoLEP for benign prostatic hyperplasia with large volume: a systematic review and meta-analysis. Am J Mens Health.

[21] Xia Z, Li J, Yang X (2021). Robotic-assisted vs. open simple prostatectomy for large prostates: a meta-analysis. Front Surg.

[22] Stuck AE, Siu AL, Wieland GD, Rubenstein LZ, Adams J (1993). Comprehensive geriatric assessment: a meta-analysis of controlled trials. Lancet.

[23] Müllhaupt G, Hechelhammer L, Graf N, Mordasini L, Schmid HP, Engeler DS, Abt D (2024). Prostatic artery embolisation versus transurethral resection of the prostate for benign prostatic obstruction: 5-year outcomes of a randomised, open-label, noninferiority trial. Eur Urol Focus.

[24] Bilhim T (2022). Long-term PAE results: what do we know. Semin Intervent Radiol.

[25] Siqueira MH, Shprits S, Bhojani N (2026). Rezūm therapy in very elderly men with BPH: two-year outcomes from a multicenter cohort. Low Urin Tract Symptoms.

[26] McVary KT, Gange SN, Gittelman MC (2016). Minimally invasive prostate convective water vapor energy ablation: a multicenter, randomized, controlled study for the treatment of lower urinary tract symptoms secondary to benign prostatic hyperplasia. J Urol.

[27] Xu M, Tao W, Zang YC, Jin L, Xue BX (2025). Efficacy of Rezūm water vapor thermal therapy system in the treatment of benign prostatic hyperplasia in high-risk elderly patients (Article in Chinese). Zhonghua Yi Xue Za Zhi.

[28] Roehrborn CG, Gange SN, Shore ND (2013). The prostatic urethral lift for the treatment of lower urinary tract symptoms associated with prostate enlargement due to benign prostatic hyperplasia: the L.I.F.T. Study. J Urol.

[29] Denisenko A, Somani B, Agrawal V (2022). Recent advances in UroLift: a comprehensive overview. Turk J Urol.

[30] Makary MA, Segev DL, Pronovost PJ (2010). Frailty as a predictor of surgical outcomes in older patients. J Am Coll Surg.

[31] Harari D, Hopper A, Dhesi J, Babic-Illman G, Lockwood L, Martin F (2007). Proactive care of older people undergoing surgery ('POPS'): designing, embedding, evaluating and funding a comprehensive geriatric assessment service for older elective surgical patients. Age Ageing.

[32] Smith R, Osterweil D, Ouslander JG (1996). Perioperative care in the elderly urologic patient. BMC Anesthesiol.

